# Deletions of Immunoglobulin heavy chain and T cell receptor gene regions are uniquely associated with lymphoid blast transformation of chronic myeloid leukemia

**DOI:** 10.1186/1471-2164-11-41

**Published:** 2010-01-18

**Authors:** Elisabeth P Nacheva, Diana Brazma, Anna Virgili, Julie Howard-Reeves, Anastasios Chanalaris, Katya Gancheva, Margarita Apostolova, Mikel Valgañon, Helen Mazzullo, Colin Grace

**Affiliations:** 1 UCL School of Medicine, Cancer Institute, Academic Haematology, Royal Free Campus, Rowland Hill Street, Hampstead, London, NW3 2PF, UK; 2 Institute of Molecular Biology, Bulgarian Academy of Sciences, Sofia, Bulgaria

## Abstract

**Background:**

Chronic myelogenous leukemia (CML) results from the neoplastic transformation of a haematopoietic stem cell. The hallmark genetic abnormality of CML is a chimeric *BCR/ABL1 *fusion gene resulting from the Philadelphia chromosome rearrangement t(9;22)(q34;q11). Clinical and laboratory studies indicate that the *BCR/ABL1 *fusion protein is essential for initiation, maintenance and progression of CML, yet the event(s) driving the transformation from chronic phase to blast phase are poorly understood.

**Results:**

Here we report multiple genome aberrations in a collection of 78 CML and 14 control samples by oligonucleotide array comparative genomic hybridization. We found a unique signature of genome deletions within the immunoglobulin heavy chain (*IGH*) and T cell receptor regions (*TCR*), frequently accompanied by concomitant loss of sequences within the short arm regions of chromosomes 7 and 9, including *IKZF1*, *HOXA7*, *CDKN2A/2B*, *MLLT3*, *IFNA/B*, *RNF38*, *PAX5*, *JMJD2C *and *PDCD1LG2 *genes.

**Conclusions:**

None of these genome losses were detected in any of the CML samples with myeloid transformation, chronic phase or controls, indicating that their presence is obligatory for the development of a malignant clone with a lymphoid phenotype. Notably, the coincidental deletions at *IGH *and *TCR *regions appear to precede the loss of *IKZF1 *and/or *p16 *genes in CML indicating a possible involvement of RAG in these deletions.

## Background

The *BCR/ABL1 *fusion, housed in the Philadelphia chromosome and resulting from t(9;22)(q34;q11) or variants, is the hallmark of chronic myeloid leukemia (CML) and the most frequent recurrent genetic aberration in B lymphoid leukemia in adults [1,2]. The *BCR/ABL1 *fusion gene encodes a constitutively active tyrosine kinase that is necessary and thought sufficient to drive malignant transformation. The natural history of the disease follows progression from a relatively benign chronic phase, through accelerated phase into terminal blast crisis. This course of events can be diverted or aborted by nearly curative therapies, such as tyrosine kinase inhibitors, transplantation and occasionally interferon [3] The staging of CML is based on clinical and pathologic features, including bone marrow and peripheral blood count, cytogenetic evolution and response to therapy rather than biological criteria. Although at present the molecular mechanisms that contribute to the development of the blast crisis are largely unknown, it is well established that the Ph bearing cells acquire additional genetic changes during disease progression [3,4]. There is evidence that the *BCR/ABL1 *fusion product not only regulates numerous proteins involved in apoptosis, proliferation and cell-cell interactions but also is a driving force behind the acquisition of additional genomic changes through regulation of the nucleotide excision/mismatch repair systems and promoting unfaithful restoration of double strand breaks [5]. This activity could lead to the formation of mutations within the *BCR/ABL1 *kinase and accumulation of additional genetic lesions, including point mutations, gene amplifications, genome loss and chromosome translocations that are believed to drive the malignant process [6,7]. Mutations or abnormal expression of *CDKN2A/2B, EVI-1, RB, MYC *and *p53 a*mong others have been reported in CML (reviewed in Radich [3]). Gene expression profiling studies have identified a relatively large number of additional genes that are differentially expressed in patients at advanced stages of the disease but no consistent pattern has as yet been established (for review see Guintas-Cardama, 2009 [4]). While recurrent genome gains at a chromosomal level, such as +8, +19, +Ph and/or iso17q, are associated with disease progression, the occurrence of translocations is infrequent[8]. The identification of cryptic deletions at der(9)t(9;22) fuelled expectations that submicroscopic lesions may mark disease evolution [9]. Indeed genome wide searches using array comparative genomic hybridization (aCGH) by us and others clearly demonstrated the accumulation of genome imbalances in advanced stages of the disease [10,11]. As the neoplastic transformation is occurring early in a haematopoietic stem cell, the resulting blast crisis (BC) in CML could be myeloid (BCM), lymphoid (BCL), or indeed mixed (BCmix). Lymphoid BC occurs in about a third of the CML patients [12] . Previous studies have shown that the BCL is characterized by the loss of *IKZF1 *in the majority of the *BCR/ABL1 *positive ALL cases and loss of *CDKN2A/B *in approximately half of the CML/BCL patients [13]. These losses were rarely present in non-BCL patients [14].

Here we report an analysis by oligonucleotide array comparative genomic hybridization (aCGH) showing the detection of universal deletions within both the immunoglobulin heavy chain and T cell receptor loci exclusively present in lymphoid blast crisis CML. These deletions are accompanied in a sub-set of cases by concomitant genome loss of regions within chromosomes 7 and 9, including the *IKZF1 *and *CDKN2A *genes, which appear to be associated with variety of structural chromosome changes - from cryptic interstitial deletions to unbalanced rearrangements resulting in short arm or whole chromosome loss.

## Results

The fluorescence ratio (FR) standard deviation (SD) for each of the 92 samples analysed with the 105 K array, ranged from 0.057 - 0.187 with a median value of 0.092. In a preliminary analysis of recurrent events, we detected a set of 1,262 loci in the BC cohort with a FR outside a threshold of ±4 SD that were seen in two adjacent probes in at least 3 samples. 410 loci corresponded to known genes after correction for copy number variations (CNV) identified by Redon *et al *[15]. These genes were found to be associated with Focal adhesion, MAPK signalling and Cell cycle control and their distribution across the genome is shown in Additional file 1: Figure S1.

### (i) Significance Analysis of Microarrays (SAM) identifies concomitant genome losses associated with lymphoid blast transformation

**Figure 1:**
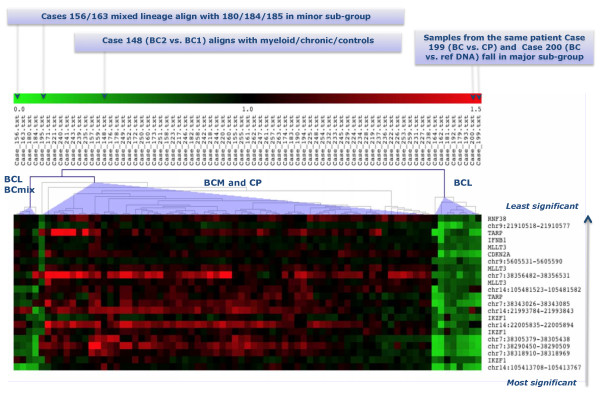
**Heat map showing the 22 most signigicant probes for the lymphoid/myeloid dichotomy identified by sam analysis**. The top 22 probes showing genome loss (in green) from a set of 435 probes identified by SAM analysis in a cohort of 60 CML and 14 control samples as significant for lymphoid blast crisis (BCL). Data fall into three clusters: on the right are 8 BCL cases, on the left are 3 lymphoid together with 2 mixed lineage (BCmix) cases, while myeloid (BCM) and chronic phase (CP) samples make up the center cluster.

We chose SAM to carry out a 2-class analysis based on myeloid and lymphoid BC CML for 74 samples - 60 CML and 14 control samples (Additional file 2: Table S1). Altogether 435 loci were found with a FDR = 0. We plotted the "expected" versus "actual" values of delta, the metric that quantifies the difference between the two classes and selected for further detailed consideration the top 22 that displayed an enhanced value of delta (Additional file 3: Figure S2). The heat map in Figure 1 illustrates the results of a hierarchical cluster analysis of these probes. The complete set of the 435 significant probes with reference to location, genome assignment and scores are given in the supplementary data (Additional file 4: Table S2). Among the known genes covered by these genome loci are *IKZF1*, *HOXA7*, *CDKN2A/2B*, *MLLT3*, *IFNA/B*, *RNF38*, *PAX5*, *JMJD2C *and *PDCD1LG2*.

**Figure 2:**
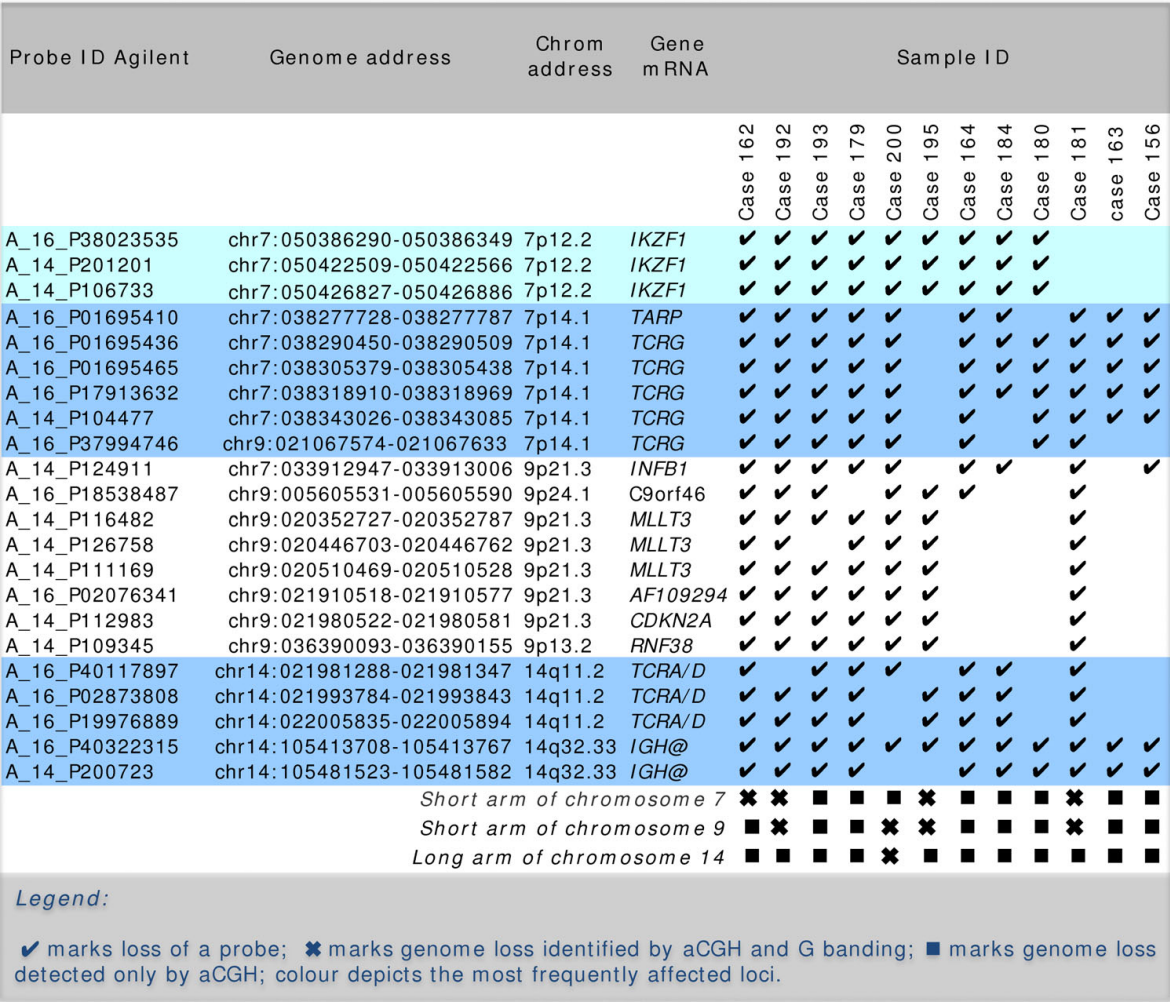
**Unique genome loss in cml/bcl samples at address 105.41 within the *IGH *region**. Graphic representation of CML/BCL samples (vertical axis) showing imbalances in the *IGH *region at 14q32.33 for the probes on the 105 K Agilent arrays (genome address shown at the bottom) with squares marking the FR values in excess of ±3 SD (calls), gains shown above and losses below with samples of chronic phase (in green), myeloid blast crisis (red) and lymphoid blast crisis (blue). Note that losses dominate the lymphoid blast samples and that the probe at 105.41 Mbp is lost in all cases.

**Figure 3:**
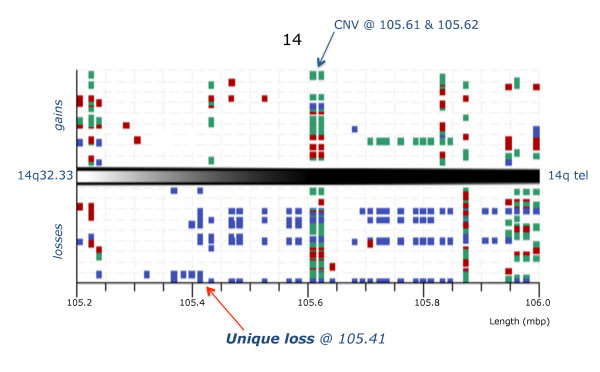
**Concomitant losses in lymphoid blast crisis of cml: top 22 genome loci**. The probe ID (Agilent), genome address, chromosome location, gene content and distribution of the losses among the 12 Lymphoid BC in the studied cohort of 78 CML samples are shown.

**Figure 4:**
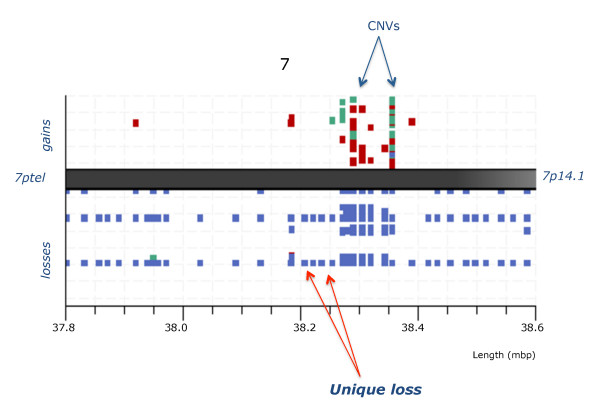
**Unique genome loss in cml/bcl samples at address 38.27-32 within the tarp/tcrg region**. Graphic representation of CML/BCL samples (vertical axis) showing imbalances in the *TARP & TCR gamma*region at 7p14.1 for the probes on the 105 K Agilent arrays (genome address shown at the bottom) with squares marking the FR values in excess of ±3 SD (calls), gains shown above and losses below with samples of chronic phase (in green), myeloid blast crisis (red) and lymphoid blast crisis (blue). Note that losses are present exclusively in the lymphoid blast samples (arrow).

The heat map (Figure 1) shows a dramatic demarcation between lymphoid/mixed lineage and the remaining samples where the green coloured columns indicate the consistent genome loss. The top 22 most significant loci are restricted to four chromosome regions, namely 7p12-14, 9p21-24, 14q11.2 and 14q32.33. Their deletion status in the 10 BCL and 2 BCmix samples is shown in Figure 2, where the probes are grouped by their genomic location. None of these probes are consistently deleted in any of the other samples studied. The probes fall into two main groups. The primary group members, highlighted in colour in Figure 2, are nearly always deleted. These include probes covering the regions of *IGH, TCR *and *IKZF1 *genes. The gains and losses for all cases in the *IGH *region are summarised in Figure 3. The locus at chr14:105.41 shows a 100% loss unique to the BCL class (in blue) whereas the loci at chr14:105.61 and chr14:105.62 display either gains or losses typical of CNVs. A region mapping to parts of the T-cell receptor gamma chain complex region (*TCR*G) extending from chr7:38.27 to chr7:38.32 on chromosome 7p14.1 is also frequently deleted (Figure 4). There are five array probes in this region, three of which are deleted in 11/12 samples (Figure 2). There are three probes in the 105 K arrays covering the *IKZF1 (Ikaros) *gene, all of which are deleted in 9/12 samples (Figure 2). The remaining probes make up the second group, which represents the full concomitant loss of loci within the 7p12-14, 9p13-24.1 and 14q11.2 regions shared by 7 of the 12 BCL samples (Figure 1 and Figure 2).

### (ii) Verification of the 105 K array results

**Figure 5:**
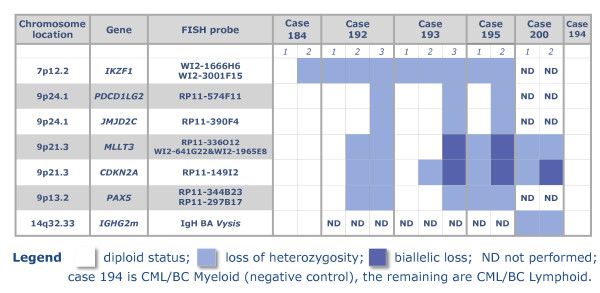
**Clonality and evolution of the concomitant 7p/9p genome loss revealed by fish**. Screening by FISH chromosome preparations from CML patients using BAC and fosmid probes confirms the aCGH data and demonstrates the progression of the concomitant loss in lymphoid blast crisis of CML.

**Figure 6:**
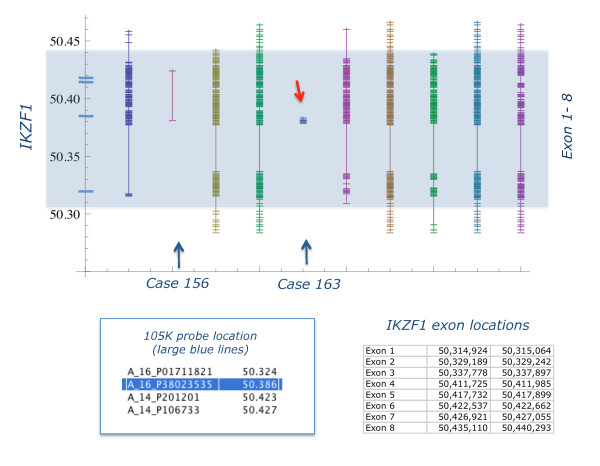
**Mapping of *IKZF1 *(IKAROS) deletions at 1 kb resolution**. Customized oligonucleotide aCGH results for *IKZF1 *(Ikaros) (exons 1-8, highlighted) for 9 CML/BCL samples. Cases 156 & 163 (arrowed) are reported normal by 105 K arrays, while the rest are deleted. The genome address is given on the left, where the large blue cross lines indicate the position of 105 K probes. Vertical lines represent results for each case - the hair lines map losses at a resolution of ~1 Kb. The two data sets are consistent for all samples but one (case 163), where the high resolution array revealed deletion of exon 3 only (red arrow), missed by 105 K array

Both FISH and aCGH on high-resolution custom oligonucleotide arrays were used to verify of the 105 K array results. FISH analysis of the *TCR *and *IGH *data was considered inappropriate due to the small size of the loci involved (2 - 50 k). All FISH tests for deletions of *IKZF1, CDKN2A, MLLT3, PAX5, JMJD2C *and *PDCD1LG2 *on cases where chromosome preparation were available were positive. Furthermore, we identified complex clonal heterogeneity as summarized in Figure 5. We applied aCGH at ~1 k resolution to the *TCR*, *IGH *and *IKZF1 *loci on 40 samples, 10 from each group of BCL, BCM, CP and controls (Figure 6 and Additional File 5: Figure S3 and Additional file 6: Figure S4). The copy number aberrations (CNA) identified by the 105 K arrays were confirmed and in particular, the deletions of *TCR *and *IGH *were shown to be remarkably consistent. These deletions along with the *IKZF1 *loss were confirmed to be exclusively present in the BCL samples as indicated by the whole genome study. Furthermore, in case 163, a deletion of 119 Kb covering only exon 3 of the *IKZF1 *region was revealed thus increasing the involvement of this gene to 10/12 BCL samples (Figure 6).

A comparison of the location of the *IGH *probes found as significant for the BCL samples with their corresponding VDJ segments within the *IGH*@ locus (IMGT database) is shown in Additional file 7: Figure S5. The probe at chr14:105.41 is located between the *IGH*J1P and *IGH*D1-26 loci, while the chr14:105.48 probe - maps between *IGH*V4-4 and *IGH*V(II)-1-1. This suggests that the observed deletions in the BCL samples are likely to result from an early VD joining that precedes any rearrangements the *IGH*@ region (Additional file 7: Figure S5). Likewise, the probes found deleted within the *TCRG *were located at the V and J segments and could reflect a recurrent rearrangement. There are three probes in the 105 K array covering the *TCR *A/D region and they actually map within the *TCR*D sequences as follows: probe chr14:21.98 is located between TRDD2 and TRDD3, chr14:21.99 is located between TRDJ1 and TRDJ4 and chr14:22.00 is located between TRDC and TRDV3 (Additional file 8: Figure S6).

### (iii) G banding and molecular karyotype differ

While the array profile of the 12 samples with lymphoid/or mixed lineage immunophenotype share the concomitant loss of loci harboured by chromosomes 7, 9 and 14, their karyotypes show no recurring pattern. Aberrations ranged from a single Ph chromosome abnormality (4/12) to multiple structural and numerical aberrations of chromosomes 7 and 9 (3/12)(Additional file 9: Table S3). Only two samples (Case 156 and 200) showed chromosome 14q32 aberrations. 4/12 samples registered deletions of chromosomes 7 and 9 consistent with their aCGH profiles. In the samples with segmental deletions losses of the short arm varied in size from 5 to 20 Mb, whilst chromosomes 7 and/or 9 are monozygous (Additional file 10: Figure S7). In cases 156, 163 (mixed lineage) and 181, the *IKZNF1 *locus was diploid whilst loci within *TARP and TCRG at 7p14.1; TCRA *at 14q11.2 and *IGH at 14q32.33 *were all deleted (Figure 2, Additional file 11: Figure S8). Case 181, which by G banding had a Ph chromosome as the sole karyotypic abnormality, was shown by array to be monozygous for the short arm of chromosome 7 with a break within 7p12.2 flanking *IKZNF1 *(which remains intact) together with a loss of the 9pter-p13.1 including the loci from the concomitant deletion (Additional file 11: Figure S8). Discrepancies between the genome profile and the chromosome findings were also recorded in Case 163 where array analysis showed a genome gain of almost the whole long arm of chromosome 17 and extra copy of an ~8 Mb region at 21q22.12 containing some of the Down Syndrome Critical regions, *ERG *and *ETS2 *among other known genes (Additional file 11: Figure S8).

### (iv) Evolution of the concomitant genome loss in BCL

**Figure 7:**
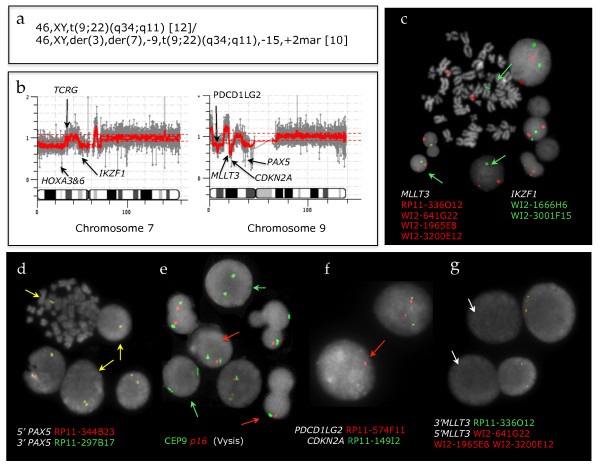
**Case 195: fish interogates discrepanicies between karyotype and acgh results**. Summary of molecular cytogenetic results for case 195: (a) G banding; (b) Array CGH (105 K Agilent) profile of chromosomes 7 and 9 with arrows pointing to sites of imbalances at the short arm regions; Representative images of cells with FISH signals showing loss of one copy of (c) *IKZF1 *(green arrows), (d) *PAX5 *(yellow arrows), (e) *p16 *(red arrows) or both copies (green arrows), (f) *PDCD1LG2 *(red arrow) and (g) bi-allelic loss of *MLLT3 *(white arrows) thus confirming the aCGH data.

FISH analysis was employed to address the discrepancies between the G banding and CGH data with main emphasis on chromosomes 7 and 9. The FISH results obtained in five cases, for which chromosome preparations were available, are summarized in Figure 5. Genome losses were confirmed in all cases studied as illustrated by the results for case 164 (Figure 7). Multiple Ph positive cell clones differing in the scope and composition of the 7p/9p regional loss were identified in 4 of the 5 cases studied ranging in complexity (Additional file 10: Figure S7). Therefore it is unlikely that the concomitant deletions in these cases result from a single event. Instead, a spectrum of consecutive aberrations probably leads to the formation of the concomitant loss.

## Discussion

Although the frequency and size of the copy number aberrations varied greatly, samples from both accelerated and blast stage CML showed substantially larger numbers of genome imbalances than chronic phase. These aberration frequencies obtained at a resolution of 33 K by oligonucleotide aCGH are broadly in agreement with earlier investigations of CML samples using BAC [10,11] and SNP array CGH [14].

Altogether 435 loci were identified as significantly different in lymphoid and myeloid BC samples. All loci were located on the short arms of chromosomes 7 & 9, with the exception of 4 from chromosomes 14, 2 from chromosome 2 and a single chromosome 22 probe. Most of the probes mapped to known genes and a number were in regions associated with immune response. We show that deletion of certain regions within *TCR *(alpha/delta and gamma) (11/12) and *IGH *(12/12), are almost universal while being accompanied by deletions within *IKZF1 *(10/12) and, to a lesser extent, *CDKN2A *and sequences within several other genes in the short arm regions of chromosomes 7 and 9 (7/12). These genome losses were not seen in any of 31 CP, 6 accelerated and 12 BCM samples or controls. Furthermore, they were present in a BCL sample (Case 199) when DNA from the patient's CP was used as reference for the aCGH analysis, thus proving their secondary origin.

A recent genome wide single nucleotide polymorphism (SNP) array analysis of Ph positive samples - 43 ALL (paediatrics and adults) and 23 CML (11 CP, 9 BCM and 3 BCL) [14] - revealed the presence of three common deletions affecting the *IKZF1, PAX5 *and *CDKN2A *loci. These deletions, confirmed by FISH and qPCR, also detected in 2 out of 3 CML BCL samples, were not always co-existent. How the reduced activity of *IKZF1 *and *PAX5 *genes, that play a key role in regulating B lineage commitment, collaborates with *BCR/ABL1 *to induce Ph(+) ALL remains unclear [14,16]. The frequency of the genome loss described by Mullighan et al.,[14] in the Ph(+) ALL, is in overall agreement with our findings in CML lymphoid blast crisis. In particular, *CDKN2A *deletions are 53.5% in Ph(+)ALL . 58.3% in CML/BCL and *PAX5 *loss is seen in 51.% of Ph(+)ALL vs. 58.3% in CML/BCL, while deletions of *IKZNF1 *are seen in 20 of 22 adults with Ph(+)ALL (90.9%) [14] and our aCGH analysis at 1 K resolution detected loss in 10/12 CML/BCL samples (83.3%). In contrast with the Ph(+) ALL results, we found that the loss of *IKZF1 *always precedes the deletions of *CDKN2A *and that losses of *RNF38 *were always accompanied by loss of the 9p13-p23.1 region that houses, among others, *CDKN2A, IFNA, RNF38, PAX5 *and *MLLT3*. In addition, we demonstrated by FISH the presence of clonal evolution leading to the concomitant 7p/9p loss as seen in less than half of patients (5/12 samples) (see typical aCGH profiles in Additional file 10: Figure S7). Importantly, the cell clone bearing the concomitant loss in the studied cases was dominant and hence, detectable by aCGH.

No imbalances of *IGH *and/or *TCR *loci were reported in Ph(+) ALL study by Mullighan et al [14] either alone or in combination with *IKZF1*, *CDKN2A *or *PAX5*. Substantial parts of the *IGH *and *TCR *regions are known to show copy number variations (CNVs) in disease free individuals http://projects.tcag.ca/variation, but whereas CNVs are characterised by amplifications and deletions of a specific locus within the sample population, we found a set of probes in the *IGH *and *TCR *regions to be universally deleted in exclusively lymphoid BC samples, thus showing features typical of secondary genomic aberrations. That they are not somatic aberrations is illustrated by Case 199, where identical features emerged when the BCL sample was hybridised against patient's DNA from the CP. These deletions characteristic of lymphoid BC were also identified in the 2 samples with mixed immunophenotype (Figure 1).

There are two possible explanations for the origin of these deletions. Either they represent genome losses associated with cross lineage rearrangements known to occur in both ALL and AML, or they represent a *bona fide *event with oncogenic potential. *IGH*/*TCR *rearrangement patterns are widely used to monitor minimal residual disease (MDR) in a range of haematological malignancies, the levels of which significantly correlate with clinical outcome [17]. The immunophenotype data for five of our 12 CML/BC samples that carry concomitant 7p/9p loss (cases 184, 192, 193, 195 & 200, Additional file 12: Table S4) are consistent with early B cell origin of the blast cells (TdT+, CD10+, CD19+, CD22+, CD7 neg, so it could be that the recurrent loss of the *TCR*G/D region in B cells with legitimate *IGH *rearrangements represents the lineage infidelity as reported previously in blast phase CML [18-21]. However, the similarity of the deletions within both immunoglobulin genes in BCL does not comply with the notion that *IGH *and *TCR *configurations are unique (clonal by origin and patient specific). The common deleted regions could indicate a preferential VDJ rearrangement in the clone that ultimately becomes dominant in the BCL patients. Such preferential VDJ usage has been reported before with a preference for the V_H_4 family segment in about 35% of children with B-ALL [22]. In our case the deletions in *IGH *and *TCR *loci possibly reflect the clonal origin of the malignancy and the stage at which the additional chromosomal aberrations that we observed were initiated. Further work needs to be done to relate these possibilities to the waterfall of changes that characterise blast crisis.

So, how does the loss of a relatively small ~100 Kb region from *IGH *and *TCR *contribute to the development of the lymphoid blast transformation? *RAG1 *and *RAG2 (RAG) *are the key components of the V(D)J recombinase machinery that catalyses the somatic gene rearrangements of antigen receptor genes during lymphocyte development. B cell progenitors undergo a development program involving ordered *IGH *gene rearrangements conducted by the RAG enzyme system. Recent evidence suggests that *BCR/ABL1 *induces expression of activation-induced cytidine deaminase (AID), found exclusively in early B cells. It participates in the class switch recombination and has been shown to lead to single strand breaks in *IGH *regions in Ph(+)ALL [23]. Therefore, it would appear that the deregulation of AID silencing by transcriptional or epigenetic factors is the key event in compromising the V(D)J recombination/RAG impairment. This could lead to the *TCR *rearrangements in B cells, which have already undergone *IGH *rearrangements and are expressing the CD10, CD19 and CD22 antigens. Thus genome losses identified in the BCL samples represent the consequence of cross lineage rearrangement i.e. DJ joining occurring in early B cells and aberrant incomplete recombination in the *TCR *complex.

This view is supported by Mullighan et al [14] who showed that the mechanism responsible for *IKZF1 *deletions in Ph(+) ALL appears to involve aberrant RAG mediated recombination as heptamer signal sequences were found internal to the deletion breakpoints. Similarly, *CDKN2A *loss was linked to RAG activity in lymphoid leukemia [24,25]. Furthermore, the impairment of the RAG system in Ph positive cells could be a direct consequence of the genetic damage caused by *BCR/ABL1 *[5,23] either through inflicting direct damage via reactive oxygen species or by compromised DNA repair [4,7]. It is also possible that the reduced/abolished expression of *c-ABL *[26] could further contribute to the impaired V(D)J recombination [27]. In summary, a chain of events initiated by *BCR/ABL1 *impairs the RAG enzyme system. The compromised V(D)J recombinase machinery is thus instrumental in creating, by means of cross lineage rearrangements, clonal populations of B-cell progenitors carrying *IGH *and *TCR *deletions.

## Conclusions

We have observed a signature of deletions that is uniquely present in our lymphoid samples. The top 22 most significant loci are restricted to four chromosome regions, namely 7p12-14, 9p21-24, 14q11.2 and 14q32.33. Their common appearance points at an event that is orchestrating the cascade of aberrations leading to the lymphoid BC. Given the high frequency of *IGH *and *TCRG *loci deletions we speculate that the evolution into lymphoid blast crisis is initiated by abnormal RAG mediated NHEJ. It appears we are edging nearer to identifying the key aberrations responsible for blast transformation at least in a proportion of the CML patients.

## Methods

### Anonymous bone marrow (BM) archival samples

from chronic myeloid leukemia (CML) patients were collected from 5 European hospitals. Altogether 92 samples were analysed of which 78 were from CML patients, 31 in chronic phase, 10 in lymphoid blast crisis, 15 in myeloid blast crisis, 2 mixed lineage acute phase, 7 unclassified and 6 accelerated phase (Additional file 2: Table S1). In addition, paired chronic and blast phase samples were available for 7 patients, of which two were classified as lymphoid blast crisis. As controls peripheral blood mononuclear cell (PBMC) samples from 11 patients with cardiovascular disease (Approval from Ethical Committee of St Anna Hospital in Sofia, Bulgaria, No 54/28.02.2005) and 3 healthy individuals (Ethical approval by NRES at Royal Free Hospital and Medical School, Ref 09/Ho720/97) were used. Cytogenetic data were available for 25 chronic and 27 blast crisis patients (Additional File 4: Table S2). Presence of the *BCR/ABL1 *fusion gene was confirmed in all samples by qPCR and/or FISH (D-FISH probe, Vysis, USA). Immunophenotyping data was available for 5 lymphoid blast phase samples (Additional file 12: Table S4).

### Array CGH and bioinformatics analysis

was performed with the Agilent 105 K oligonucleotide arrays (Wilmington, DE, USA) following the manufacturer's protocol. 500 ng genomic test DNA was extracted from either peripheral blood or BM samples. Sex mismatched pooled DNA from PBMC of 6 - 8 disease free individuals (Promega, UK) was used as reference in all but the seven CML patients where the matched CP and BC samples were hybridized against each other. Anonymous DNA samples from PBMC of patients with cardiovascular disease and healthy individuals were used as controls. The dye swapped arrays were scanned at 5 μm resolution and the resulting data analyzed using GenePix v6 software http://www.moleculardynamics.com and MIDAS v2.19 http://www.tigr.org. We chose to analyse the results on the basis of the fluorescence ratio (FR) calculated as the normalized ratio of test to reference DNA, using the simple ratio rather than the logarithmic ratio. We used an iterative method to calculate a standard deviation undistorted by outliers for the purposes of establishing a preliminary measure of aberration complexity threshold. The emergence of high throughput technology such as microarrays raises a fundamental statistical issue relating to testing hundreds of hypotheses thus rendering the standard P value meaningless [28]. Instead, the False Discovery Rate (FDR) concept is becoming the key statistical assessment tool replacing the P value. Tusher et al [29] described such a method, Significance Analysis of Microarrays (SAM) and the implementation due to Chu et al [30] has been incorporated by TIGR into their suite of 'MeV' routines. SAM uses permutations of the repeated measurements to estimate the FDR, the percentage of genes identified by chance. We used SAM with a FRD = 0 for the supervised analysis of the myeloid and lymphoid blast crisis, chronic and control samples. Firstly, after removing data for the sex chromosomes, we constructed a table defining the FR for each locus and assigned classes e.g. blast crisis (BC), chronic phase (CP) and Control; Lymphoid or Myeloid; Male or Female. We chose a two class unpaired test using myeloid vs. lymphoid immunophenotype, and applied SAM to ask if there were any probes that were uniquely associated with either blast cell lineage. All genome addresses are derived from build 35 (March 2006) of the Human Genome.

### A Customized oligonucleotide array CGH

comprising 8 × 15 k probe sets per *slide *was manufactured by Agilent to our specification and used to verify the losses identified by the standard whole genome 105 K array. Oligonucleotide probes from Agilent were selected to cover regions at ~1 k intervals except from genome locations where the presence of repetitive sequences disallowed the design of reliable probes. The arrays were scanned, features extracted and the data analysed using an Agilent scanner and DNA Analytics software. High-resolution graphics were generated using Mathematica^® ^http://www.wolfram.com.

### FISH mapping

was carried out for further verification of the aCGH results to identify the genome aberrations at a cellular level using Bacterial Artificial Chromosomes (BAC) clones obtained from the BACPAC Resources Center (Children's Hospital Oakland Research Institute, Oakland, CA, USA), the Sanger Centre (Cambridge, U) and *Invitrogen *(Paisley, UK) following protocols in routine use [31]. Where possible a minimum of 100 interphase and 50 metaphase cells were screened with a dual colour dual FISH assay using BAC/fosmid probes selected for optimal coverage of the regions of I *KZF1, CDKN2A, MLLT3, PAX5, PDCD1LG2 *and *JMJD2C *genes. The size of the FISH probes used was in the range of 200 - 400 Kb.

## Authors' contributions

EN wrote the grant application and the manuscript, designed and supervised the study; DB performed array CGH and FISH experiments; JHR, AV, MV and HM contributed to G banding and FISH investigations; AC contributed to the analysis of the results and helped draft the manuscript; MA, KG carried out aCGH tests of the control samples and CG undertook the aCGH/SAM analysis and co-wrote the manuscript. All authors read and approved the final manuscript.

## Supplementary Material

Additional file 1 **Figure S1**. Active genome loci in cml. Click here for file

Additional file 2 **Table S1**. Id number, sex and disease stage of the 92 cml samples. Click here for file

Additional file 3 **Additional Figure 2**. Observed vs expected values of delta (Δ) for ~100,000 probes. Click here for file

Additional file 4 **Additional Table 2**. Annotated flourescence ratio data for 105 k arrays: Listing of probes, locations, gene assignments, lineage, gender and sample identifiers. Click here for file

Additional file 5 **Figure S3**. Mapping of igh deletions at average 1 kb resolution. Click here for file

Additional file 6 **Figure S4**. Mapping of tcr gamma deletions at verage 1 kb resolution. Click here for file

Additional file 7 **Figure S5**. THE *IGH *GENE COMPLEX. Click here for file

Additional file 8 **Figure S6**. The *TCRG *gene complex. Click here for file

Additional file 9 **Table S3**. Immunophenotype and karyotype data for 27 samples from patients in blast crisis of cml. Click here for file

Additional file 10 **Figure S7**. Representative array cgh profiles of concomitant loss of chromosomes 7 and 9 in cml lymphoid blast crisis. Click here for file

Additional file 11 **Figure S8**. Representative array cgh profiles of cml/bcl cases with intact ikaros. Click here for file

Additional file 12 **Table S4**. Sample data for 5 cases with lymphoid blast crisis. Click here for file
