# Supplementary figures and images for: Deletions of Immunoglobulin heavy chain and T cell receptor gene regions are uniquely associated with lymphoid blast transformation of chronic myeloid leukemia
Source: BMC Genomics. 2010 Jan 18;11:41. doi: 10.1186/1471-2164-11-41 (PMC2822760; doi:10.1186/1471-2164-11-41)

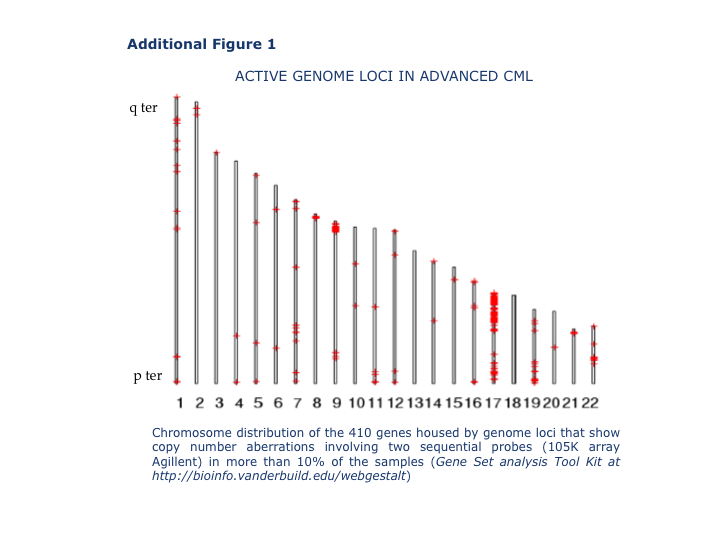

Supplement: Additional file 1 — Figure S1. Active genome loci in cml. [file 1471-2164-11-41-S1.PNG]

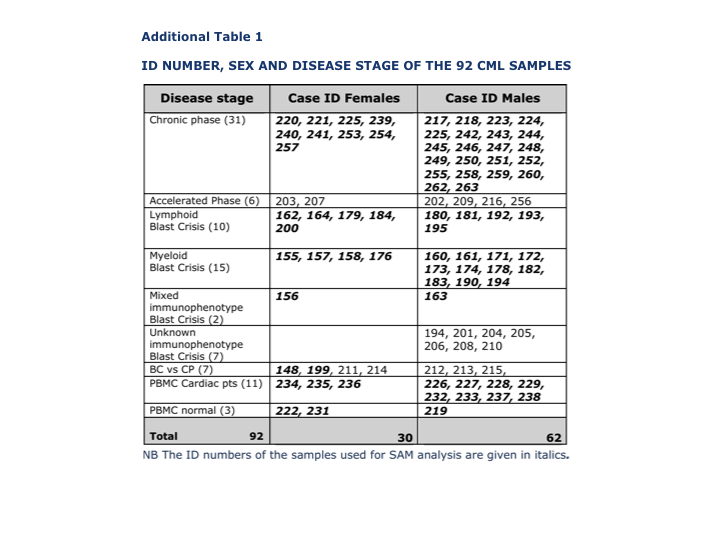

Supplement: Additional file 2 — Table S1. Id number, sex and disease stage of the 92 cml samples. [file 1471-2164-11-41-S2.PNG]

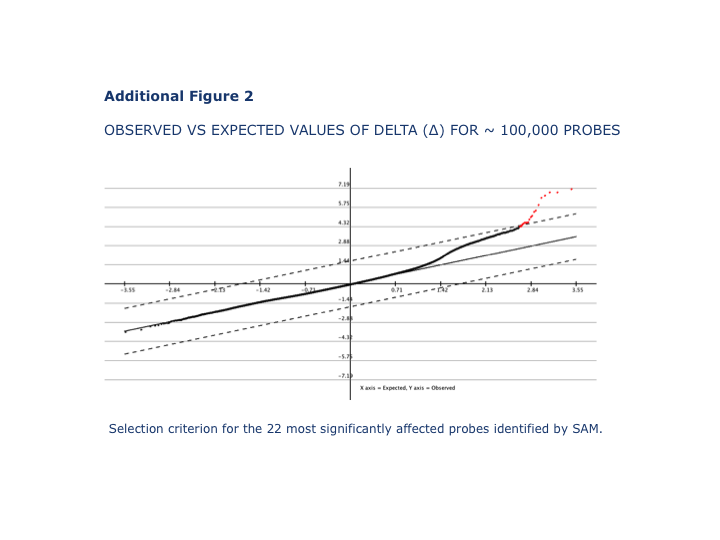

Supplement: Additional file 3 — Additional Figure 2. Observed vs expected values of delta (Δ) for ~100,000 probes. [file 1471-2164-11-41-S3.PNG]

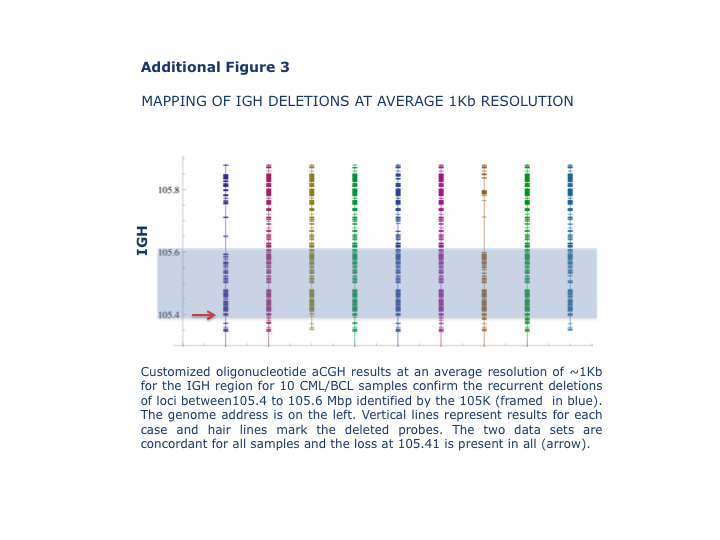

Supplement: Additional file 5 — Figure S3. Mapping of igh deletions at average 1 kb resolution. [file 1471-2164-11-41-S5.PNG]

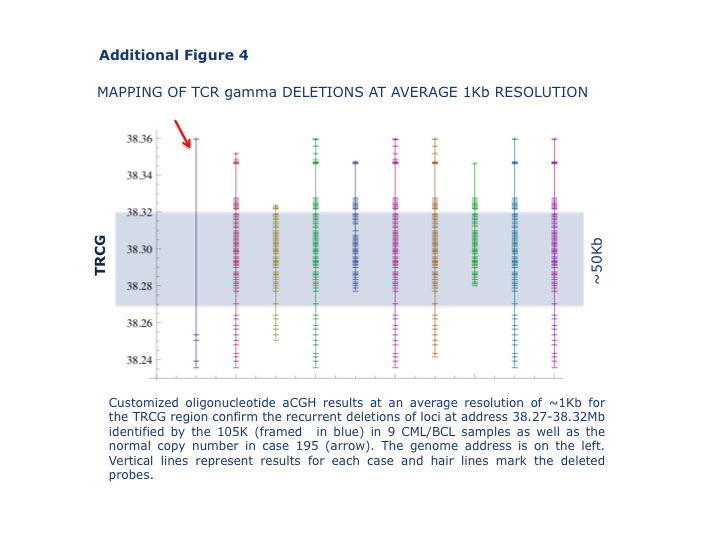

Supplement: Additional file 6 — Figure S4. Mapping of tcr gamma deletions at verage 1 kb resolution. [file 1471-2164-11-41-S6.PNG]

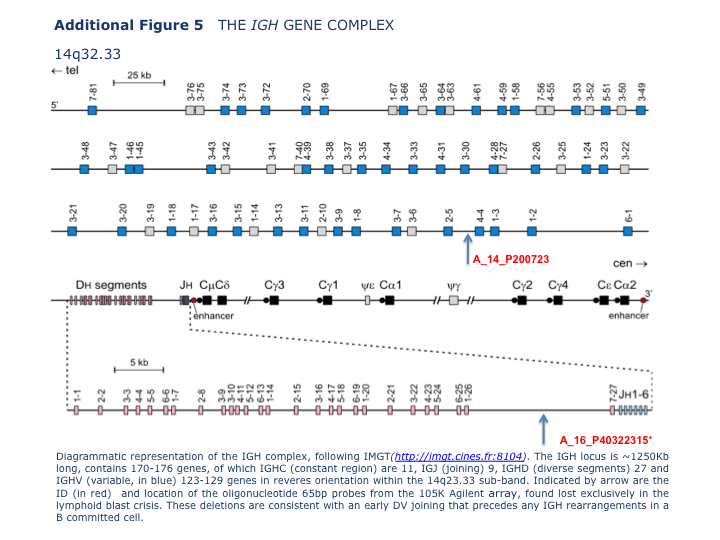

Supplement: Additional file 7 — Figure S5. THE IGH GENE COMPLEX. [file 1471-2164-11-41-S7.PNG]

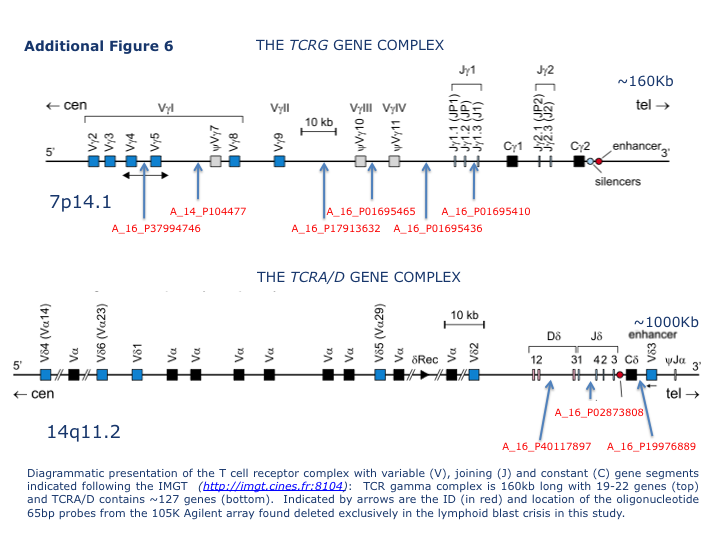

Supplement: Additional file 8 — Figure S6. The TCRG gene complex. [file 1471-2164-11-41-S8.PNG]

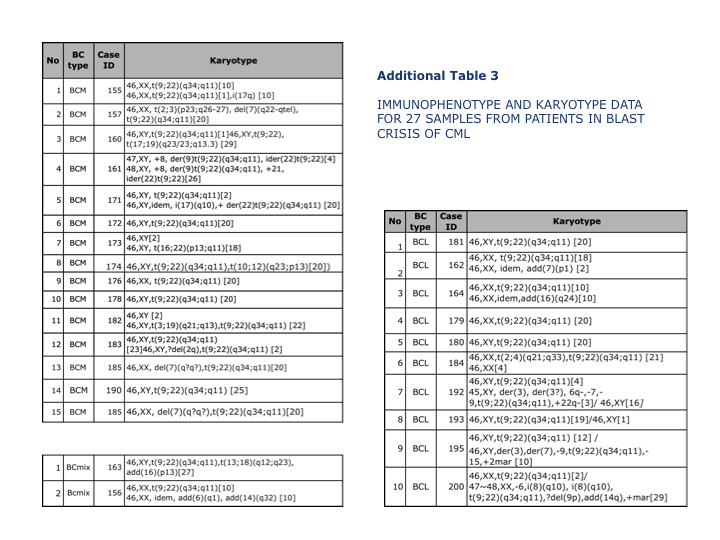

Supplement: Additional file 9 — Table S3. Immunophenotype and karyotype data for 27 samples from patients in blast crisis of cml. [file 1471-2164-11-41-S9.PNG]

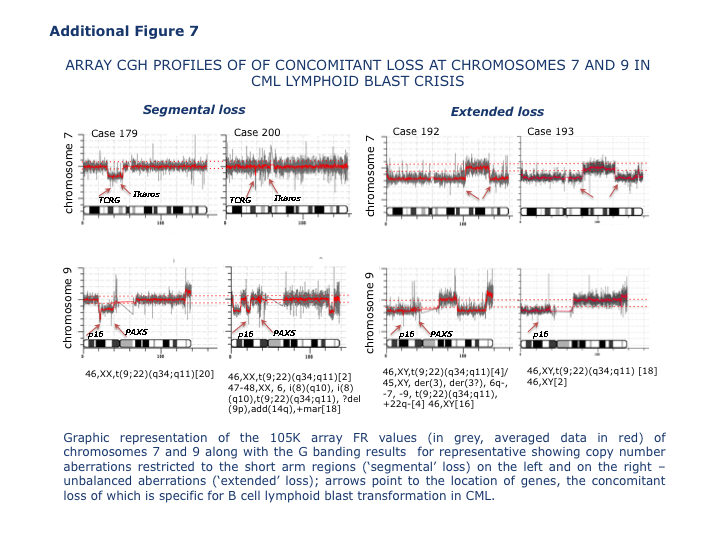

Supplement: Additional file 10 — Figure S7. Representative array cgh profiles of concomitant loss of chromosomes 7 and 9 in cml lymphoid blast crisis. [file 1471-2164-11-41-S10.PNG]

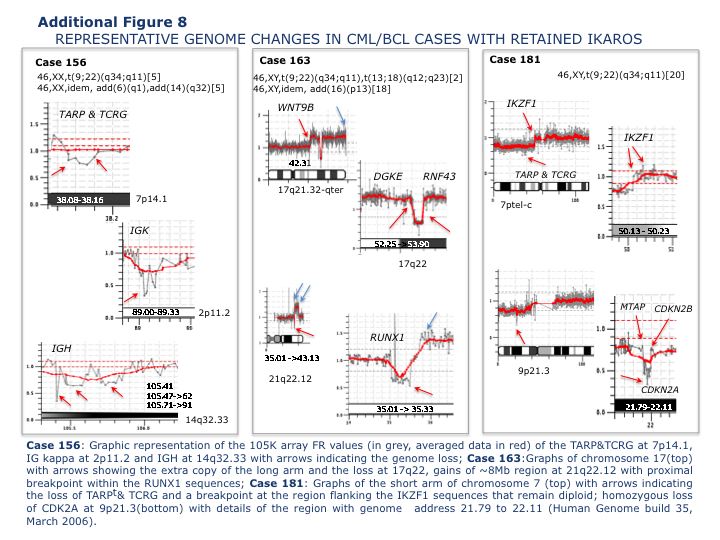

Supplement: Additional file 11 — Figure S8. Representative array cgh profiles of cml/bcl cases with intact ikaros. [file 1471-2164-11-41-S11.PNG]

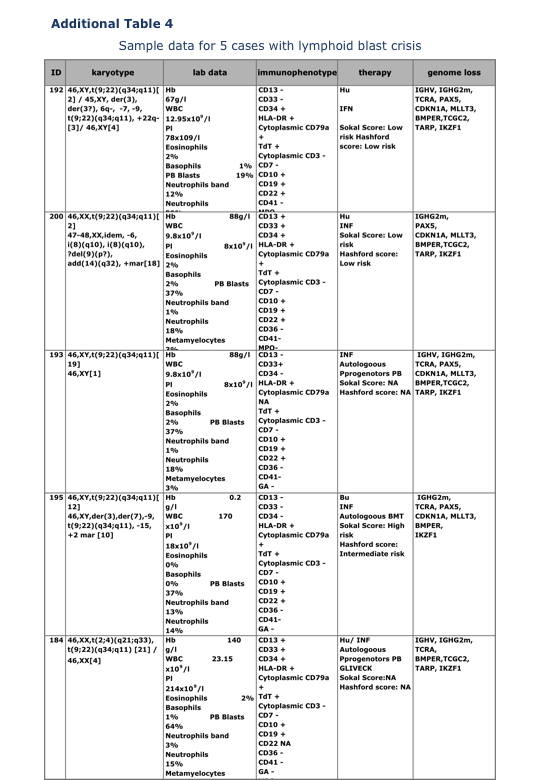

Supplement: Additional file 12 — Table S4. Sample data for 5 cases with lymphoid blast crisis. [file 1471-2164-11-41-S12.PNG]
